# Effect of community active case-finding strategies for detection of tuberculosis in Cambodia: study protocol for a pragmatic cluster randomized controlled trial

**DOI:** 10.1186/s13063-020-4138-1

**Published:** 2020-02-24

**Authors:** Alvin Kuo Jing Teo, Kiesha Prem, Konstantin Evdokimov, Chetra Ork, Sothearith Eng, Sovannary Tuot, Monyrath Chry, Tan Eang Mao, Li Yang Hsu, Siyan Yi

**Affiliations:** 1 Saw Swee Hock School of Public Health, National University of Singapore and National University Health System, Singapore, Singapore; 2 Department of Infectious Disease Epidemiology, Faculty of Epidemiology and Population Health, London School of Hygiene and Tropical Medicine, London, UK; 3 KHANA Center for Population Health Research, Phnom Penh, Cambodia; 4 Cambodia Anti-Tuberculosis Association, Phnom Penh, Cambodia; 5 National Center for Tuberculosis and Leprosy Control Cambodia, Phnom Penh, Cambodia; 6 Yong Loo Lin School of Medicine, National University of Singapore and National University Health System, Singapore, Singapore; 7 Center for Global Health Research, Touro University California, Vallejo, CA USA; 8 School of Public Health, National Institute of Public Health, Phnom Penh, Cambodia

**Keywords:** Active case finding, Passive case finding, Systematic screening, Community, Tuberculosis

## Abstract

**Background:**

Cambodia has made notable progress in the fight against tuberculosis (TB). However, these gains are impeded by a significant proportion of undiagnosed cases. To effectively reach people with TB, active case-finding (ACF) strategies have been adopted by countries affected by the epidemic, including Cambodia, alongside passive case finding (PCF). Despite increased efforts to improve case detection, approximately 40% of TB cases in Cambodia remained undiagnosed in 2018. In Cambodia, several community-based TB ACF modalities have been implemented, but their effectiveness has yet to be systematically assessed.

**Methods:**

This pragmatic cluster randomized controlled trial will be conducted between December 2019 and June 2021. We will randomize eight operational districts (clusters) in seven provinces (Kampong Cham, Kampong Thom, Prey Veng, Thbong Khmum, Kampong Chhnang, Kandal, and Kampong Speu) to either the control group (PCF) or the intervention groups (ACF using a seed-and-recruit model, ACF targeting household and neighborhood contacts, and ACF targeting persons aged ≥ 55 years using mobile screening units). The primary endpoints will be TB case notification rates, additionality, and cumulative yield of TB cases. The secondary endpoints include treatment outcomes, the number needed to screen to find one TB case, and cost-effectiveness outcome measures. We will analyze the primary and secondary endpoints by intention to treat. We will compare cluster and individual-level characteristics using Student’s *t* test and hierarchical or mixed-effect models to estimate the ratio of these means. The incremental cost-effectiveness ratio per disability-adjusted life year averted will also be considered as a benchmark to determine whether the interventions are cost-effective.

**Discussion:**

This study will build an evidence base to inform future scale-up, implementation, and sustainability of ACF strategies in Cambodia and other similar settings. Implementation of this study will also complement TB control strategies in Cambodia by conducting ACF in operational districts without active interventions to find TB cases currently. Those who are ill and might have TB will be promptly screened, diagnosed, and linked to care. Early diagnosis and treatment initiation will also benefit their community by interrupting transmission and prevent further infections. The experience gained from this project will inform future attempts in conducting pragmatic trials in low-resource settings.

**Trial registration:**

ClinicalTrials.gov, NCT04094350. Registered on 18 September 2019.

## Introduction

### Background and rationale

Cambodia is one of the countries with a high tuberculosis (TB) burden, with an estimated incidence of active TB of 302 (95% CI: 169–473) per 100,000 population in 2018 [1]. Over the years, the country has made notable progress in the fight against TB. In 2016, the TB incidence was approximately half of that in 2000, and a similar decline was observed in the TB mortality rate [2]. Cambodia also recorded a TB treatment success rate of 94%, one of the highest among the 30 countries with a high TB burden [3]. However, the successes are encumbered by a significant proportion of undiagnosed cases. Globally, it is estimated that 30% of TB cases were undiagnosed in 2018 [1]. Similarly, approximately 40% of TB cases goes undetected in Cambodia [2]. Each missing case continues to perpetuate the transmission of TB and contribute to the current TB burden, compounding the challenge to end TB. Houben and Dodd estimated that 60% of Cambodia’s population in 2014 were infected with latent TB [4]. As the population ages over time, it is projected that the prevalence of active TB will increase [5]. Therefore, it is pertinent to reach TB-affected individuals and ensure accountable and effective TB treatment to break the cycle of TB transmission. Acceleration of efforts to find TB cases and narrowing the gap in diagnosis is one way to reduce the TB burden in Cambodia [5].

Traditionally, TB cases are reported and passively notified when people with TB present themselves to a health facility. Active case finding (ACF), a proactive TB case-finding approach, has been adopted by countries affected by the epidemic, including Cambodia, to effectively reach people with TB [6–8]. Current evidence suggests that TB case-finding interventions at the community level may increase TB case detection [8]. In Zimbabwe, Corbett et al. demonstrated that ACF using a mobile van approach resulted in a higher yield of smear-positive TB cases compared to ACF conducted via door-to-door visits [9]. The prevalence of smear-positive TB also declined significantly during the intervention period, suggesting that ACF could interrupt TB transmission [9]. In Vietnam, a household-contact investigation was reported to be more effective than standard passive case finding alone for the detection of TB [10]. However, despite increased efforts to improve case detection, integrating TB case finding in health-care systems remains a great challenge in diverse socioeconomic, geographical, epidemiologic, and other sociocultural contexts [11, 12].

Cambodia has achieved a significant reduction in TB burden through patient-centered approaches, strong community mobilization, and decentralization of TB care to the primary level of health care to reach underserved communities [13, 14]. Within the rubric of community TB care, the national TB program (NTP) has implemented several successful models of TB case-finding initiatives in collaboration with civil society organizations. Under a community TB screening approach, household and other close contacts of people with smear-positive TB were referred to health centers and screened using a TB symptoms questionnaire and chest X-ray (CXR). This approach has been shown to increase TB case notifications by 65% compared to historical baseline cases in the preceding year [7]. Economic analyses have also suggested that this approach is cost-effective across a range of parameters such as numbers of people screened and the yield of TB cases [15]. In another model, one-off ACF using mobile diagnostic units (GeneXpert and CXR) focused on people aged ≥ 55 years has also been reported to increase the yield of TB diagnoses and improve treatment outcomes [16].

To further reduce the TB burden, efforts should be strengthened through systematic engagements with groups at disproportionate risk for TB [17]. The NTP in Cambodia has since recalibrated its focus to direct community TB activities at key affected populations (KAPs) to accelerate TB case detection. In the Cambodian context, the KAPs for TB identified by the NTP are people aged 55 years and older, people living with HIV, close contacts of people with smear-positive TB, prisoners, people with diabetes, and people who use drugs [18]. To effectively reach KAPs for TB, a community-based snowball approach (seed-and-recruit mechanism) has been recently implemented. Empirically, a peer-led approach as such has been widely accepted to reach concealed populations, such as populations who are at risk for HIV in many countries, including Cambodia, due to its practical feasibility, and successes have been reported [19–21]. The seed-and-recruit model, piloted in 2018 in Cambodia, is well received and deemed feasible by the beneficiaries and key stakeholders [22]. Preliminary analyses of the effectiveness of the model showed a 97% increase in the yield of TB diagnoses (unpublished data; Ly C, personal communication, June 5, 2019) compared to historical baseline in the previous year.

At present, little is understood about how to best apply and integrate ACF in the existing health-care systems in diverse epidemiologic, socioeconomic, and cultural contexts [12]. In Cambodia, the different ACF strategies—community seed-and-recruit mechanism, contact investigations and referrals, one-off roving ACF using mobile diagnostic tools—have been empirically shown superior to passive case finding (PCF), but the effectiveness of these approaches in improving TB case detection has yet to be systematically assessed. Moreover, there is still insufficient evidence on the effect of ACF on treatment [8] and the cost-effectiveness outcomes of ACF in different settings [11].

### Objectives

The primary aim of this study is to evaluate the effectiveness of ACF strategies in increasing the yield of TB diagnoses and case notifications in Cambodia. This study also aims to establish the effect of ACF strategies on TB treatment outcomes and to establish the number needed to screen to detect one TB case. Lastly, this study aims to estimate the cost-effectiveness of the interventions by measuring the cost per TB case identified and the incremental cost-effectiveness ratio.

### Trial design

**Fig. 1:**
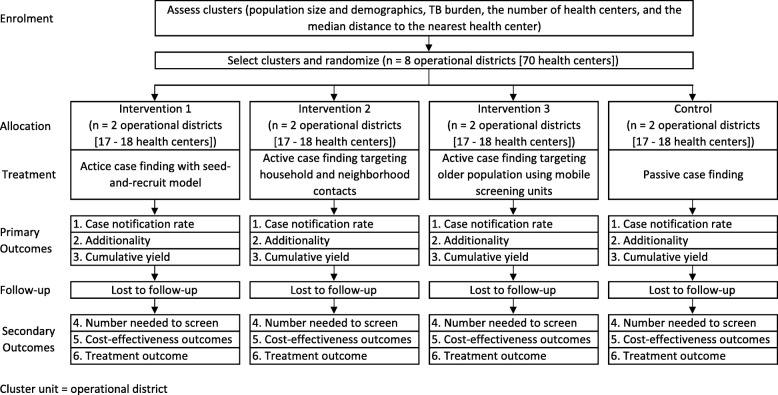
Trial profile. A pragmatic cluster randomized controlled trial with four arms comparing active case finding (ACF) with the seed-and-recruit model, ACF targeting household and neighborhood contacts, ACF targeting the older population using mobile diagnostic units, and passive case finding in eight operational districts in Cambodia. *TB* tuberculosis

We will conduct a pragmatic cluster randomized controlled trial with four arms comparing ACF with a seed-and-recruit model, ACF targeting household and neighborhood contacts, ACF targeting the older population using mobile screening units, and a PCF approach in eight operational districts (ODs) in Cambodia (Fig. 1). These case-finding strategies have been piloted in Cambodia, and the interventions will be carried out as per the protocol devised by the partner organizations, respectively. Target populations and presumptive TB cases will be evaluated clinically, with CXR and/or with bacteriological testing of sputum using either smear microscopy or the GeneXpert system, according to the national TB guideline [23]. A pragmatic cluster design was chosen because the interventions will be conducted at the community (collective) level in a “real-world” setting for the avoidance of contamination, and it is not practically possible to randomize the interventions at an individual level.

## Methods: participants, interventions, and outcomes

### Study setting

The sampling frame consisted of operational districts (ODs) without ACF interventions currently, and in the preceding 6 months from the project implementation date. Using data from the Demographic Epidemiological Model of Cambodia developed in collaboration with the National Center for Tuberculosis and Leprosy Control (CENAT) [5] and other health and demographic data from Cambodia [24, 25], eight ODs (Fig. 1) have been purposively selected to include ODs with high and low incidence to maintain as much balance as possible. The selection was also based on the number of health centers to increase the comparability and generalizability of study findings across the groups.

### Eligibility criteria

In this study, an individual will be defined as presumptive TB if he/she exhibits any of the following symptoms [26]:

- Pulmonary TB: a cough for more than 2 weeks and at least one general symptom
- Extra-pulmonary TB: presence of symptoms, depending on the location of TB (e.g., enlarged lymph node, swollen backbone, swollen articulation, etc.), and at least one general symptom
- General symptoms: fever, night sweats for more than 2 weeks, or unintentional weight loss (> 5% reduction in usual body weight over the last 6–12 months) [27]

Presumptive TB cases will be referred to the health centers for TB screening and diagnosis in the intervention arms and self-presented to the health centers in the control arm. We will include the aggregated number of cases diagnosed and notified from all arms regardless of age. However, we will only recruit people aged 18 years or older with TB (all forms) for the baseline and follow-up surveys and assessment of TB treatment outcomes.

### Who will take informed consent?

Data collectors (project staff from Cambodian Anti-Tuberculosis Association (CATA), KHANA, CENAT, and/or health center staff) will be trained to explain the study to the participants, obtain written consent for the surveys (Additional file 2), and administer the questionnaire. The data collectors will brief participants on the nature and purpose of the study and potential risks, and will highlight that information will be kept confidential. Consent will be taken by the data collectors in a private space at the respective OD after randomization. For participants who cannot read or write, verbal consent will be taken, and this will be noted by the consent taker on the consent form.

### Additional consent provisions for collection and use of participant data and biological specimens

No biological samples will be collected for research purposes; hence, no additional consent will be sought from the participants. Biological samples will be collected as part of standard care (e.g., TB diagnosis and case management) defined in the national TB guideline under the purview of the NTP.

### Interventions

#### Explanation for the choice of comparators

The PCF strategy is a default setup in the national health system. PCF is a patient-initiated pathway that relies on the self-presentation of presumptive TB cases to the health centers to be diagnosed with TB. Upon screening by health center staff, sputum samples from presumptive TB cases belonging to the KAPs will be collected for examination by GeneXpert or direct smear microscopy if the GeneXpert system is not available. CXR will be performed at the referral hospital if the sputum examination result is negative. TB diagnoses will be made by clinicians based on clinical, radiological, and microbiological evidence. For presumptive TB individuals who present with cough for less than 2 weeks, CXR will be performed at the referral hospital, and sputum samples will be collected for GeneXpert testing if radiological abnormalities are detected [23]. For presumptive TB cases who do not belong to KAPs, smear examination will be performed if presenting with a cough lasting for 2 weeks or more. Those presenting to the health centers with cough for less than 2 weeks will be referred to the referral hospital for CXR, and cases with any radiological abnormalities will undergo smear examination [23]. Newly diagnosed people with TB will be treated and managed according to the national TB guidelines. We will follow-up all people with TB diagnosed during the study period for 6 months from the treatment initiation.

This is a patient-initiated pathway to TB diagnosis involving: a person with active TB experiencing symptoms that he or she recognizes as serious; a person having access to and seeking care, and presenting spontaneously at an appropriate health facility; a health worker correctly assessing that the person fulfills the criteria for presumptive TB; and the successful use of a diagnostic algorithm with sufficient sensitivity and specificity to diagnose TB.

#### Intervention description

##### Intervention 1: ACF with the seed-and-recruit model

**Fig. 2:**
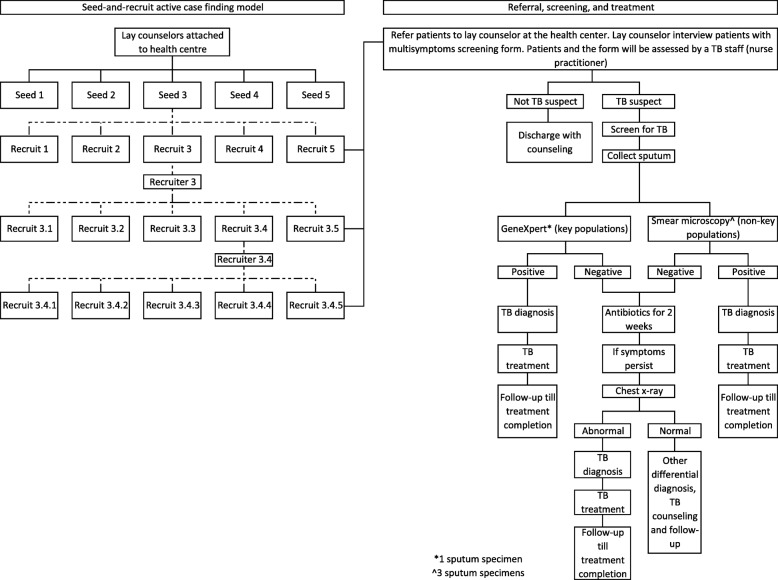
Active case finding with a seed-and-recruit model. A network is built by seed, as illustrated by the dotted lines in the left panel. The network is expanded in a snowball fashion by recruiters who will be trained to recruit other people who might have tuberculosis (TB) in the community. We refer key populations for TB in this study to people aged 55 and above, people with diabetes, people living with HIV, household contacts of TB patients, and people who use and inject drugs. For GeneXpert test, one sputum specimen will be collected. For smear microscopy, three sputum specimens will be collected at three different times, over 2 days

In the intervention clusters, a lay counselor appointed to a health center will act as focal point for identifying potential seeds (TB survivors and their family members and other key informants in the community, such as moto-taxi drivers and grocery store owners), screening presumptive TB cases using a screening questionnaire, and training seeds and recruiters to find other presumptive TB cases in the community. Each lay counselor will be tasked to identify at least five seeds per health center to identify presumptive TB cases (recruits) in their community. These recruits will be screened for TB at the health centers and linked to care if TB is diagnosed. Among the new diagnoses, those who are eligible (Fig. 2) will be trained to become recruiters to find and refer other presumptive TB cases to the health centers in a snowball approach. The research team will work with staff at the health centers to facilitate screening and enrollment of recruits who are diagnosed with TB to care. Eligibility criteria for lay counselors, seeds, and recruiters are illustrated in Additional file 1.

All presumptive TB cases referred by seeds and recruiters will be screened by lay counselors using a symptoms screening questionnaire [17]. The screening for symptoms compatible with TB includes a cough lasting for at least 2 weeks, hemoptysis, fever, weight loss, and night sweats [17]. Presumptive TB cases and their symptoms screening questionnaire will be assessed by a nurse practitioner in the health center for further TB work-up. Those who are presumed to have TB will be asked to submit sputum samples at the health center for acid-fast bacilli examination. Sputum samples of KAPs will be assessed using the GeneXpert system at the referral hospital laboratory. For non-KAPs, direct smear microscopy will be undertaken at the nearest laboratory. If the sputum examination results are negative, further assessments and diagnosis of TB will be made on clinical and radiological grounds by clinicians. TB screening and treatment will be provided at the health facilities at no cost to the patient, in accordance with the policies of the NTP. Cases suspected of extrapulmonary TB (presence of local and general symptoms [23]) will be referred to the OD referral hospital for further assessment and diagnosis. We will follow-up all people with TB referred by seeds for 6 months from the treatment initiation.

##### Intervention 2: ACF targeting household and neighborhood contacts

**Fig. 3:**
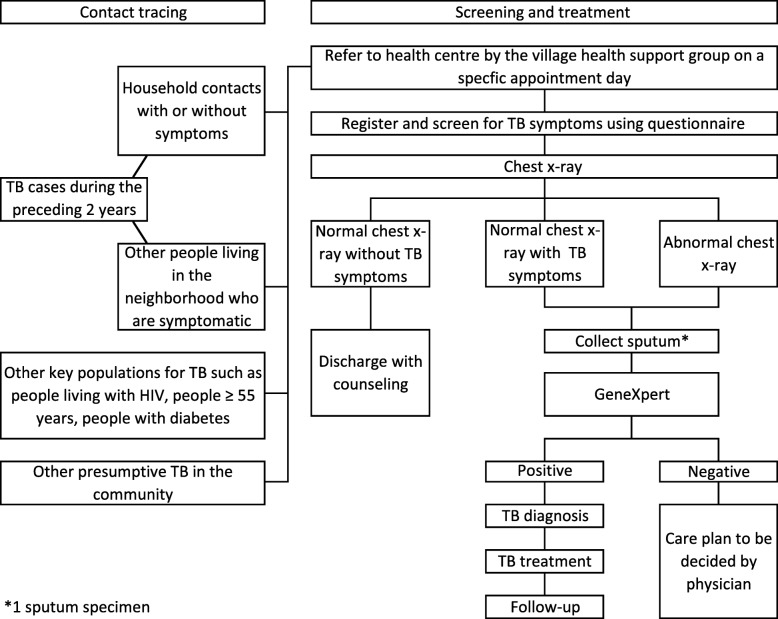
Active case finding targeting household and neighborhood contacts. This model targets household and neighborhood contacts of people with TB. The village health support group will conduct contact tracing and case finding activities in the community before inviting the target groups to the health center for TB screening and diagnosis. For GeneXpert test, one sputum specimen will be collected. *HIV* human immunodeficiency virus, *TB* tuberculosis

In this arm (Fig. 3), community health volunteers will recruit household contacts of people with TB and TB survivors diagnosed in the preceding 2 years. Immediate neighbors (10 nearest households) of the index cases (people with TB) who are symptomatic will also be invited by the community health volunteers to the screening session. Other KAPs for TB and presumptive TB cases in the community encountered by the community health volunteers will also be invited to the screening session.

The one-off screening session will be held at all of the health centers in the selected ODs on specific days. Presumptive TB cases will be screened for TB symptoms on-site, and CXR will be taken. Sputum samples from presumptive TB cases exhibiting features suggestive of TB (on CXR and/or presence of TB symptoms) will be collected for GeneXpert testing. Test results will be communicated to the newly diagnosed people with TB, and they will be referred to the health centers for treatment and follow-up. Should the health center of their choice not fall within the selected sites, follow-up (for the follow-up survey) will be conducted via telephone calls. Cases suspected for extrapulmonary TB will be either diagnosed on-site or referred to the referral hospital for further assessment if needed. We will follow-up on all cases found in this arm for 6 months from the start of TB treatment.

##### Intervention 3: ACF targeting the older population (people aged ≥ 55 years) using mobile screening units

**Fig. 4:**
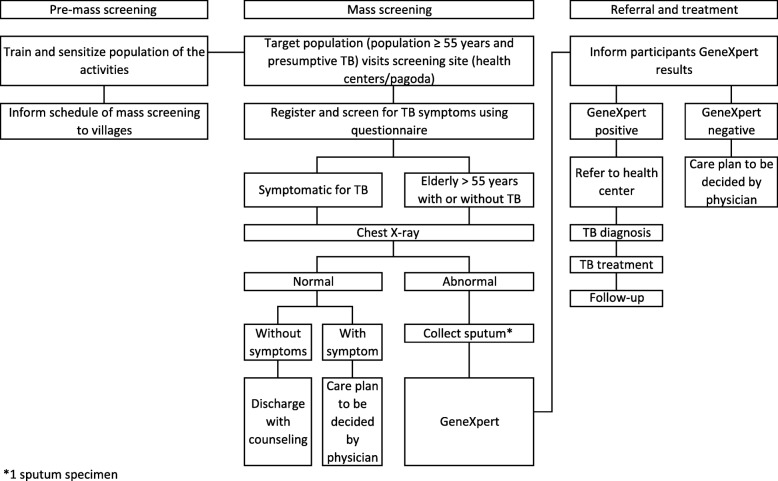
Active case finding targeting the older population (people aged 55 and above) using mobile screening units. In the pre-mass screening stage, program staff will invite the target population (people aged 55 and above) and other presumptive TB cases in the community to a roving, one-off active case finding day at the health centers or other public sites in the community such as the pagoda. For GeneXpert test, one sputum specimen will be collected. *TB* tuberculosis

In the intervention clusters (Fig. 4), an outreach team will conduct training and sensitization of the target population prior to the screening session. We will set up a screening site at a designated time, day, and place. Communities in the districts will be informed of the schedule for a one-off mass screening session before the screening day. Each person who visits the screening session will be screened and surveyed at registration by trained staff. Information on their age, demographics, and presence of TB symptoms will be collected. CXR will then be performed on-site for all persons exhibiting TB symptoms, and all individuals aged ≥ 55 years regardless of symptoms. CXR will be assessed by trained CXR readers on-site. When features suggestive of TB are found, sputum samples will be collected for GeneXpert testing on-site as well. Test results will be communicated to the participants immediately or via telephone calls, and people with TB will be referred for treatment and follow-up at the health center where screening is conducted or a health center of their choice. TB treatment and care at the health center will be provided according to the guidelines of the NTP. Should the health center of their choice not fall within the selected sites, follow-up will be conducted via telephone calls. Cases suspected for extrapulmonary TB will be referred to the referral hospital for further assessment and diagnosis. A follow-up will be conducted 6 months from treatment initiation. The standard operating protocol of mobile screening sessions depicts that the intervention will only be conducted at the selected sites once in 1–3 years.

#### Criteria for discontinuing or modifying allocated interventions

Since the interventions implemented in this study will be part of standard procedures endorsed by the NTP in Cambodia, the activities will not adversely affect the welfare of the participants and pose no more than minimal risk. Therefore, we do not foresee discontinuation or modification of allocated interventions. In this study, participation in the activities will be voluntary and the participants can withdraw from the study at any point in time.

#### Strategies to improve adherence to interventions

In this study, the implementing organizations will design and ensure that the interventions’ (TB case-finding) protocols are strictly adhered. The medical procedures and laboratory tests (e.g., GeneXpert, CXR, and sputum microscopy) conducted in this study are in accordance with the national TB guidelines. In addition, the National University of Singapore, which is not an implementing organization, will conduct regular field visits and interim analyses to ensure adherence to study protocol.

### Relevant concomitant care permitted or prohibited during the trial

No concomitant care would be specifically permitted or prohibited during the trial due to the pragmatic nature of the study. Interventions will be conducted at the community level according to standard operating procedures in a “real-world” setting.

### Provisions for post-trial care

Post-trial care will be provided in accordance with the standard care as defined in the national TB guidelines under the purview of the NTP.

### Outcomes

We will measure the following endpoints in all four arms. The primary endpoint will be the case notification rate in the intervention and control ODs during the study period. We define a TB case to be either bacteriologically confirmed (smear-positive identified by smear microscopy, culture, or rapid diagnostics approved by the World Health Organization (WHO)), smear-negative TB (two sputum samples tested negative and clinically diagnosed by a clinician—with abnormal CXR or no improvement in response to a course of broad-spectrum antibiotics), or extrapulmonary TB (presence of local symptoms such as enlarged lymph nodes, joint swelling, and Gibbus deformity and presence of general symptoms such as cough, fever, weight loss, and night sweats) [23]. This will be ascertained from the results of the screening and routine case notification data reported to the NTP. We will also determine: additionality, comparing the yield in each arm with its respective historical baseline (same period in the preceding year); and cumulative yield over the project implementation period. Secondary endpoints will include: the number of people needed to screen to detect one TB case; direct and indirect costs per TB case notified; and the treatment outcome of all people with TB in this study. These data will be collected on the aggregated form at the health center level or as reported by the project implementation team in each arm, except cost data, which will be collected from study participants in the baseline and follow-up surveys.

### Participant timeline

The participant timeline is presented in Table 1.

**Table 1:** Schedule of enrollment, implementation of interventions, assessments, and data collection for the period of the trial

Timepoint	Study period								
	Enrollment	Allocation	Post-allocation						
	2019	2019	2019	2020	2020	2020	2020	2021	2021
	Q3	Q3	Q4	Q1	Q2	Q3	Q4	Q1	Q2
Enrollment (cluster level)									
Assessment of cluster	X								
Randomization of cluster	X								
Allocation of intervention		X							
Interventions									
Active case finding with a seed-and-recruit model			X	X	X	X	X		
Active case finding targeting household and neighborhood contacts			X	X	X	X	X		
Active case finding targeting people aged 55 years and older using mobile screening units			X	X	X	X	X		
Passive case finding			X	X	X	X	X		
Assessments (cluster level)									
TB case notification by selected clusters			X	X	X	X	X		
TB cases reported by each arm			X	X	X	X	X		
Individuals screened for TB			X	X	X	X	X		
Treatment outcomes					X	X	X	X	X
Cost data			X	X	X	X	X	X	X
Assessments (individual level)									
Enrollment			X	X	X	X	X		
Baseline survey			X	X	X	X	X		
Follow-up survey					X	X	X	X	X

*TB* tuberculosis, *Q* quarter

### Sample size

Simulations were conducted to estimate the power of the trial to detect a statistically significant difference between the intervention arms and the control arm for the primary outcome of the trial. To develop a basis for these simulations, we examined the ranges and variability of effect sizes from previously collected data that included operations data from CATA, CENAT, KHANA, and the NTP annual TB report 2018 [28]. We defined effect size as the percentage increase in the number of cases notified to the NTP because of the intervention (baseline: PCF). CATA, CENAT, and KHANA have implemented the respective interventions (different strategies of ACF) at different sites in Cambodia between 2017 and 2018. The percentage of estimated additional cases notified to the NTP by CATA, KHANA, and CENAT during the intervention period (scenario 1) was 110%, 98%, and 30%, respectively. For this scenario, the power was over 90%. We also considered three more sets of conservative effect sizes: (scenario 2) assuming all intervention arms have an effect size of 75%; (scenario 3) assuming all intervention arms have an effect size of 50%; and (scenario 4) assuming all intervention arms have an effect size of 25%. There was a similar level of power for a more cautious estimate (scenario 2—a 75% improvement in case notification). In scenarios 3 and 4, the power was maintained above 70% that a difference between intervention and control arms could be detected.

### Recruitment

The trial will be conducted at the selected ODs, and all communities and individuals who are eligible will be invited to participate in the activities.

### Assignment of interventions: allocation

#### Sequence generation

The unit of randomization will be the ODs (clusters). From the list of underserved ODs in Cambodia provided by CENAT, we will randomly allocate four ODs with high TB incidence and four ODs with low TB incidence to the intervention and control arms, matched by the population size and the number of health centers.

#### Concealment mechanism

Randomization will be performed by a person who will not be otherwise engaged in the project and will be blinded to the identity of the districts. Project coordinators and residents in the selected ODs will not be masked to the intervention. All case-finding activities share the same objective—to find TB cases—and they will be done without reference to the intervention group.

#### Implementation

The population living in the selected ODs will be included in the respective cluster. There will be four arms (Fig. 1), and two clusters will be randomly allocated to each arm. As all interventions have been endorsed by the CENAT and conducted in Cambodia, participants will not be notified if they are part of the intervention or the control group.

### Assignment of interventions: blinding

#### Who will be blinded

The data analysts will be masked to intervention allocation and will only analyze de-identified data.

#### Procedure for unblinding if needed

This pragmatic cluster randomized trial is open-label, where project coordinators and staff are not blinded. All case-finding activities will be implemented in the selected sites, as indicated. Study participants and health center/laboratory staff will not be notified if they are part of the intervention group or the control group. Therefore, unblinding will not occur in this trial.

### Data collection and management

#### Plans for assessment and collection of outcomes

For the primary endpoints, we will collect case notification data from the intervention implementers (CATA, KHANA, CENAT), the provincial health department, and health centers (Table 2). For the secondary endpoints, data on treatment outcomes will be collected from the health centers. To assess the number needed to screen, we will collect screening data and the number of TB cases diagnosed from the intervention implementers, the provincial health department, and health centers. Data on costs—direct and indirect medical costs [29, 30], intervention, diagnostics, and medications—will be collected from the intervention implementers and the baseline and follow-up surveys (Table 2). In the survey, we will document the baseline characteristics of new diagnoses found in all arms using a questionnaire. Information on age, gender, education level, occupation, number of family members and housing, smoking, alcohol use, drug use, TB history, and the presence of other comorbidities will be collected. We will adapt and contextualize the knowledge, attitude, and practice survey instrument tools developed by the WHO to collect information on TB knowledge, attitudes, and care-seeking behavior toward TB [31]. A short follow-up survey will be conducted at the end of the TB treatment period for each person with TB to collect information on missed doses, side effects, and other TB treatment-related costs incurred.

**Table 2:** Endpoints and data collection

Study endpoint	Description	Data source
Primary endpoints		
Case notification rates (cases notified per 10,000 population per year)	Numerator: number of cases notified by the selected districts	Case notification data from CENAT
	Denominator: total population in the OD	Population statistics (Ministry of Planning/Department of Statistics/CENAT)
Additionality (additional number of cases reported compared to historical baseline)	Number of cases notified by the selected districts	Case notification data from CENAT
	Historical data (cases notified in the preceding 3 years)	Historical case notification data from CENAT
Cumulative yield (cases diagnosed per 1000 screened)	Numerator: number of cases reported by each arm	Program data In the control arm, data to be collected from the health centers monthly
	Denominator: total number of individuals screened	Program data Provincial health department laboratory data to determine the number of people screened at health centers in the control arm
Secondary endpoints		
Treatment outcomes	TB treatment outcomes of all new patients 6 months after treatment initiation	Health centers
Number needed to screen	Numerator: total number of individuals screened	Program data Provincial health department laboratory data to determine the number of people screened at health centers in the control arm
	Denominator: number of cases reported by each arm	Program data Control arm: data to be collected from the health centers monthly
Cost per TB case diagnosed/notified	Direct and indirect medical costs while seeking care for TB Staff and intervention costs Diagnostics and medication costs	Health-care-seeking costs: data to be collected in the baseline and follow-up survey Intervention costs: data to be collected from KHANA, CENAT, and CATA DALYs: data to be extracted from WHO global burden of disease studies and other existing literature
Incremental cost-effectiveness ratio per DALY averted		

*CATA* Cambodia Anti-Tuberculosis Association, *CENAT* National Center for Tuberculosis and Leprosy Control, *DALY* disability-adjusted life year, *OD* operational district, *TB* tuberculosis, *WHO* World Health Organization

Data collectors (project staff from CATA, CENAT, KHANA, and/or health center staff) will be trained to explain the study to the participants, obtain written consent for the surveys (Additional file 2), and administer the questionnaire. The data collectors will brief participants on the nature and purposes of the study and potential risks, and will highlight that information will be kept confidential. Consent for the surveys will be taken by the data collectors in a private space after randomization.

Data collectors will administer the questionnaire (both questionnaire 1—baseline survey and questionnaire 2—follow-up survey) on-site using a tablet-administered format. The baseline survey will take between 30 and 40 min to complete, and the follow-up survey will take approximately 30 min to complete. The surveys may be conducted via telephone calls for participants who are unable to visit the health centers for interviews. Refusals will be excluded from this study, but consent will be sought to record demographic characteristics (participants would only be completing the “Socio-Demographics” section of the questionnaire if they have signed the Consent Form indicating consent to participate in the study but later choose not to complete the questionnaire). Participants will be reimbursed USD 2 for their participation at baseline (questionnaire 1) and USD 2 for an exit interview (questionnaire 2) at the end of the 6-month follow-up.

#### Plans to promote participant retention and complete follow-up

The plans to promote participant retention and to manage refusals are illustrated in the previous section “Plans for assessment and collection of outcomes”.

#### Data management

Data coding, quality control, and data entry will be done following established procedures at KHANA and Saw Swee Hock School of Public Health, National University of Singapore. A database will be developed for data entry in KoBoToolbox [32], which includes a built-in range, and within and between-variable consistency checks. The program also runs error-checking applications and produces reports of inconsistencies to be checked daily. The database will be exported into Excel to check for consistency and to clean before data analyses. All data will be entered into the database within 1 month of collection. For baseline data, a precoded questionnaire will be used to minimize data coding errors. All data forms and questionnaires will be checked for errors by the field supervisors, and necessary corrections will be made during data entry.

#### Confidentiality

The steps taken to maintain participants’ confidentiality during the consent-taking process are illustrated in the earlier section “Who will take informed consent?”. Furthermore, only project staff from CATA, CENAT, and KHANA will have access to the personal data in the trial. These data will not be published, and they will be discarded after the publication of results.

#### Plans for collection, laboratory evaluation, and storage of biological specimens for genetic or molecular analysis in this trial/future use

This trial will evaluate four models of case detection to reach people with TB who are undiagnosed effectively. The collection and evaluation of sputum samples and other biological specimens in the course of this trial for the diagnosis and management of TB will be conducted following the standard protocol and guidelines in Cambodia [23]. It is not within the scope of this study to conduct additional analyses on the biological specimens collected. Hence, the management of biological specimens collected in the course of this trial will be under the purview of the NTP as they would normally be handled outside the research.

### Statistical methods

#### Statistical methods for primary and secondary outcomes

For the primary endpoints, we will compute TB case notification at the cluster level by dividing the total number of TB cases (all forms) notified by the selected ODs by the total population in each OD. Case notification rates will be presented as TB cases notified per 100,000 population per year. The difference in the total number of TB cases notified during the intervention period and the number of TB cases notified in the preceding 3 years will be computed to determine additionality. We will calculate the cumulative yield of TB cases by dividing the number of cases notified in each arm by the total number of people screened for TB. Cumulative yield will be presented as TB cases notified per 1000 individuals screened.

For the secondary endpoints, we will evaluate the treatment outcome of TB cases detected in this study according to the following categories: treatment completed (without evidence of failure); treatment failed (sputum smear remains positive at month 5 or later of treatment); died (death due to any reasons before and during treatment); lost to follow-up (people newly diagnosed with TB who do not initiate treatment or treatment course that is disrupted for 2 or more successive months); not evaluated (no treatment outcome data available); and cured (sputum smear converted from positive to negative in the final month of the treatment and on at least one earlier assessment at month 2 or month 5) [23]. The number needed to screen to detect one TB case in each arm will be calculated by dividing the total number of persons screened by the number of TB cases detected (i.e., the reciprocal of the absolute cumulative yield). Direct and indirect medical costs that will be incurred before and during health-seeking will be measured based upon the baseline and follow-up questionnaires. Costs for identification of presumptive TB cases and engagement with KAPs for screening, including training and meetings before initiating ACF activities, will be categorized separately from the costs for diagnosis using radiological/bacteriological means and management of people with TB. The incremental cost-effectiveness ratio (ICER) will be estimated by dividing the difference in total costs between trial arms by the difference in the total number of TB cases detected. The ICER per disability-adjusted life year averted will also be considered as a benchmark to determine whether the interventions are cost-effective.

We will analyze the primary and secondary endpoints by intention to treat, and the analyses will be conducted in two stages. First, we will analyze and present primary and secondary endpoints using the cluster summary method. The baseline characteristics of all arms at the cluster level and individual-level characteristics at baseline and endline will be compared using Student’s *t* test and hierarchical or mixed-effect models to estimate the ratio of these means. As the ODs have several health centers, we will adopt a linear mixed-effects model adjusting for baseline measurements to account for clustering in the data. Individual-level data will also be incorporated into multivariable regression models to account for potential confounders differing by the trial arm and improve the precision of the risk ratios by accounting for intercluster variations. The impact of TB case-finding strategies on treatment outcomes will be evaluated using survival analysis to compare treatment success rates across the intervention and control arms. The study will be reported according to the Consolidated Standards of Reporting Trials (CONSORT) [33] statement (Additional file 3), the template for intervention description and replication (TIDieR) [34] checklist for intervention description and replication (Additional file 4), the Standard Protocol Items: Recommendations for Interventional Trials (SPIRIT) [35] statement (Additional file 5), and the Pragmatic Explanatory Continuum Indicator Summary-2 (PRECIS-2) [36] for pragmatic trials (Additional file 6). All statistical analyses will be conducted using STATA 14 (Stata Corp LP, TX, USA) and R (R Foundation for Statistical Computing, Vienna, Austria).

#### Interim analyses

Interim analyses will be conducted by the data analyst who is otherwise masked to intervention allocation.

#### Methods for additional analyses (e.g., subgroup analyses)

The baseline characteristics of participants in all arms at the cluster level and individual-level characteristics at baseline and endline will be compared using Student’s *t* test and hierarchical or mixed-effect models to estimate the ratio of these means. As the ODs have several health centers, we will adopt a linear mixed-effects model adjusting for baseline measurements to account for clustering in the data. Individual-level data will also be incorporated into multivariable regression models to account for potential confounders differing by the trial arm and improve the precision of the risk ratios by accounting for intercluster variations.

#### Methods in analysis to handle protocol nonadherence and any statistical methods to handle missing data

We will adopt intention-to-treat analyses, and imputation methods will be considered in the event of missing data.

#### Plans to give access to the full protocol, participant-level data, and statistical code

Information from the full protocol will be published in a peer-reviewed journal. The relevant data analyzed during the development of this study protocol are available upon request from the corresponding author.

### Oversight and monitoring

#### Composition of the coordinating center and trial steering committee

The coordinating center will be based in the KHANA Center for Population Health Research. The steering committee will comprise representatives from CATA, CENAT, KHANA, and Saw Swee Hock School of Public Health, National University of Singapore. Data will be managed by the team at KHANA, with inputs from the other two implementing organizations. The research team from Saw Swee Hock School of Public Health, National University of Singapore will be responsible for the analyses of the data from the trial. The steering committee will adjudicate the endpoints and develop the findings dissemination plan in consultation with the data monitoring committee.

#### Composition of the data monitoring committee, its role, and the reporting structure

The data monitoring committee (DMC) will comprise representatives from KHANA Center for Population Health Research and Saw Swee Hock School of Public Health, National University of Singapore. The DMC will be formed independently from the funders. The DMC will be responsible for the independent assessment of the validity and integrity of the pragmatic cluster randomized controlled trial. Interim analyses will be presented to the DMC. The DMC will report trial progress and the results of the trial steering committee. The DMC will meet twice a year, or more if needed.

#### Adverse event reporting and harms

There are no major perceivable risks in this study. The potential psychological distress because of the questions that the study poses is minimal. Participation is purely voluntary, and the decision to not participate in this study or to terminate their participation at any point during the study will not have any negative consequences. All participants and those who refuse to partake in the survey will not be deprived of the standard care they ought to receive for TB. As the interventions we are evaluating are operations that have been implemented and endorsed by the NTP, the risks have been thoroughly deliberated. The interventions involve only models of care to reach people with TB. TB screening, diagnosis, and treatment algorithms included in these models are per standard protocol in Cambodia.

#### Frequency and plans for auditing trial conduct

The research team will conduct at least two monitoring visits to each OD during the 1-year implementation period. Field supervisors will also conduct at least four monitoring visits (e.g., every quarter for KHANA arms where the intervention is spread over 1 year and at every screening session conducted by CATA and CENAT during the implementation period, respectively). During monitoring visits, we will review consent forms, completeness of the data collection forms, and compliance with the trial protocol. Any anomalies in recruitment and data will be followed up by the field staff. Interim analyses will be conducted by the data analyst who is otherwise masked to intervention allocation. Data, recruitment processes, and interim results will be monitored by the research team and reported to the DMC.

#### Plans for communicating important protocol amendments to relevant parties (e.g., trial participants, ethical committees)

Amendments to the protocol will be duly communicated to all relevant parties, including the NTP and all partners, investigators, ethics boards in Cambodia and Singapore, the trial registry, and journals.

#### Dissemination plans

We will share the findings from this study in results dissemination meetings with key stakeholders and peer-reviewed journals. Findings from this trial could also potentially inform ACF strategies in other countries with a high TB burden.

## Discussion

In partnership with CATA, CENAT, and KHANA, this pragmatic cluster randomized controlled trial seeks to examine the effect of community-based ACF strategies in increasing TB case detection and its impact on treatment outcome. This trial focuses on the ACF approaches, which are currently adopted as one of the mainstay strategies to promote early TB diagnosis in Cambodia [37, 38]. We will undertake a pragmatic approach [39] by testing existing interventions against usual care (PCF) in a setting where these interventions have been previously conducted to enhance generalizability and replicability in Cambodia and other similar settings in the future.

KAPs at risk for TB, such as people living with HIV, close contacts of people with TB, and persons aged 55 years and older [18], are often those with limited access to health care, leading to a delay in TB diagnosis and poor clinical outcome [40] and further transmission of the infection in the community [41]. Therefore, the prioritization of high-risk groups for systematic screening of TB has been identified as a key component in the global efforts to end TB [26]. However, ACF activities can be costly [42], and a rigorous assessment of cost-effective ACF strategies is therefore a critical justification for TB control program planning and scale-up in a high-burden, often resource-poor setting. Also, the implementation of this project will complement TB control programs in Cambodia by expanding ACF to ODs that are currently not served by CENAT, and other implementing organizations, thus rendering operation and strategic benefits to the country’s efforts to find undiagnosed TB cases in the community. It will also contribute to the capacity-building of local staff in research.

An important foreseen limitation of our study is that we will not be able to determine the impact of ACF on the prevalence of TB in Cambodia. However, we believe that the primary and secondary endpoints of this study will generate an evidence base that is relevant to Cambodia and generalizable beyond this research setting.

This project is congruent with the global plan to end TB [43] by informing sustainable and evidence-based solutions for TB control in Cambodia. Upon completion of this trial, the results will inform and enable a nationwide scale-up of an effective intervention that is contextualized and complies with the principles set by the NTP to find undiagnosed cases and control TB in Cambodia.

## Trial status

Protocol version 1, December 3, 2019. Recruitment began on December 18, 2019 and will be completed by approximately December 31, 2020.

## Supplementary information

**Additional file 1.** Supplementary material.

**Additional file 2.** Participant information sheet.

**Additional file 3.** CONSORT 2010 checklist of information to include when reporting a cluster randomized trial.

**Additional file 4.** The TIDieR (Template for Intervention Description and Replication) checklist.

**Additional file 5.** SPIRIT 2013 Checklist: recommended items to address in a clinical trial protocol and related documents.

**Additional file 6.** PRECIS-2 scores for trial domains.

## Data Availability

Data sharing is not applicable to this article as no datasets were generated or analyzed during the current study. However, any data required to support the development of this protocol can be supplied upon request.

## Abbreviations

ACF: Active case finding
CATA: Cambodia Anti-Tuberculosis Association
CENAT: National Center for Tuberculosis and Leprosy Control
CI: Confidence interval
CXR: Chest X-ray
DMC: Data monitoring committee
HIV: Human immunodeficiency virus
ICER: Incremental cost-effectiveness ratio
KAP: Key affected population
NIHA: National University of Singapore Initiative to Improve Health in Asia
NTP: National tuberculosis program
OD: Operational districts
PCF: Passive case finding
TB: Tuberculosis
USD: US dollar
WHO: World Health Organization
