# Supplementary material for: Effect of community active case-finding strategies for detection of tuberculosis in Cambodia: study protocol for a pragmatic cluster randomized controlled trial
Source: Trials. 2020 Feb 24;21:220. doi: 10.1186/s13063-020-4138-1 (PMC7041270; doi:10.1186/s13063-020-4138-1)
Supplement: Supplementary file 1 — Additional file 1. Supplementary material. [file 13063_2020_4138_MOESM1_ESM.docx]

**Additional file 1: Supplementary Material**

Eligibility criteria of lay counselors

1. Able to read and write
2. Residing in the community where work is carried out
3. Have had TB and been cured
4. Has good relationship with the community authorities and health centers
5. Has own transport

Eligibility criteria of seeds and recruiters

1. Newly diagnosed TB patients who are undergoing treatment
   1. Know TB symptoms
   2. Volunteers to find other presumptive TB in the community
2. Non-TB patients but belonging to these population groups – family of people living with TB, moto-taxi drivers, grocery store sellers
   1. Know TB symptoms
   2. Know 5 or more presumptive TB in their network
