# Supplementary material for: Effect of community active case-finding strategies for detection of tuberculosis in Cambodia: study protocol for a pragmatic cluster randomized controlled trial
Source: Trials. 2020 Feb 24;21:220. doi: 10.1186/s13063-020-4138-1 (PMC7041270; doi:10.1186/s13063-020-4138-1)
Supplement: Supplementary file 2 — Additional file 2. Participant information sheet. [file 13063_2020_4138_MOESM2_ESM.docx]

# Participant Information Sheet

You are invited to participate in a research study. This information sheet provides you with information about the research study. The Principal Investigator (the research doctor or the person in charge of this research) or his/her representative will also describe this research to you and answer all of your questions. Read the information below and ask questions about anything you don’t understand before deciding whether or not to take part.

**PART I: General Information**

1. **Protocol title**

Effect of community active case finding strategies for detection of tuberculosis in Cambodia: a pragmatic cluster randomized controlled trial

1. **Principal Investigator and co-investigator(s), if any, with the contact number and address of organization**

Dr Siyan Yi

*Senior Research Fellow*

Saw Swee Hock School of Public Health

Tahir Foundation Building, National University of Singapore

12 Science Drive 2, Singapore 117546

[ephsyi@nus.edu.sg](mailto:alvin.teo@u.nus.edu)

Phone: (65) 6516 4988

**Co-investigators:**

Associate Professor Hsu Li Yang

Head of the Infectious Disease Programme

Saw Swee Hock School of Public Health

Tahir Foundation Building, National University of Singapore

12 Science Drive 2, Singapore 117546

[mdchly@nus.edu.sg](mailto:mdchly@nus.edu.sg)

Dr Konstantin Evdokimov

*Research Fellow*

Saw Swee Hock School of Public Health

Tahir Foundation Building, National University of Singapore

12 Science Drive 2, Singapore 117546

[ephke@nus.edu.sg](mailto:ephke@nus.edu.sg)

Mr Alvin Teo Kuo Jing

*PhD Student*

Saw Swee Hock School of Public Health

Tahir Foundation Building, National University of Singapore

12 Science Drive 2, Singapore 117546

[alvin.teo@u.nus.edu](mailto:ephke@nus.edu.sg)

1. **Whom should I call if I have any questions or problems?**

Please contact the Principal Investigator, *Siyan Yi* or Attn: *Tuot Sovannary* at **telephone** +855 12 836 926 and **email** *ephsyi@nus.edu.sg* for all research-related matters and in the event of research-related injuries.

This study has undergone an ethics review by the National Ethics Committee for Health Research Cambodia and the National University of Singapore Institutional Review Board (NUS-IRB). For an independent opinion regarding the ethics of this research and the rights of research subjects, or if you have complaints about the research, you may contact a staff member of the NUS-IRB (Attn: Dr Chan Tuck Wai, at telephone (+65) 6516 1234 [Mondays to Thursdays from 8.30am to 6pm, and Fridays from 8.30am to 5.30pm, except public holidays] or email at irb@nus.edu.sg).

1. **Who can participate in the research? What is the expected duration of my participation? What is the duration of this research?**

To take part in the research, you must be 18 years old and above. Those who do not fulfill the inclusion criteria as stated above and refuse to participate will be excluded. The survey will take approximately 30 to 40 minutes to complete.

1. **What is the approximate number of research subjects involved?**

A total of 2000 participants like you will be invited to participate in this study, which involves answering a questionnaire.

1. **What will be done if I take part in this research study?**

You are required to answer a questionnaire. The questionnaire is divided into four sections. The interviewer will ask you questions in section 1 to 3 at baseline. A few additional questions in section 4 will be asked 6 months from the date of treatment initiation. The baseline survey will take 30 to 40 minutes, and the survey at the 6^th^ month will take 30 minutes to complete.

We will ask you a few demographic questions, your medical and family history, and the onset of TB symptoms at baseline. We will also ask questions regarding your knowledge and perception of TB, use of health services, practices of prevention and control methods of TB, and healthcare-seeking costs. We will also collect clinical data from the health center’s medical records on your TB diagnosis, HIV status, if available, which is part of the routine procedures in the health center. You will be given time to read and understand the information provided in this sheet. You can choose to take part in the study after you have understood the purpose and procedure of the study. In addition, you may stop at any time during the survey, and if there are questions that you would prefer not to answer, you do not have to.

1. **Will there be reimbursement for participation?**

A token of appreciation will be given for your time and inconvenience in participating in this study. If you complete the baseline survey, you will be reimbursed USD 2. The reimbursement will not be pro-rated if you withdraw from the interview midway.

1. **How will my privacy and the confidentiality of my research records be protected?**

Research data will be coded, and we will treat your responses with confidentiality. The database will be kept confidential and will not be made publicly available. No one will have access to the database except the research team. Your name and contact information will only be used to contact you for surveys, and it is only available to the research staff. Your address will also be obtained in the reimbursement acknowledgment sheet which would be forwarded to the NUS Finance Department. Your personal data will be discarded after the publication of the results. Consent forms which bear your name will be stored for 10 years. It will not be used in the analysis, and publications/presentation related to this study. All research data collected will be kept in accordance with the National University of Singapore’s Research Data Management Policy. Research data (without personal identifiers) used in any publication will be kept for a minimum of 10 years before being discarded. Where any Personal Data is collected from you, we will keep the information confidential in accordance with the Human Biomedical Research Act and other applicable legal rules.

1. **What is the nature of this biomedical research?**

This survey will be conducted within the context of a cluster randomized trial to evaluate the effectiveness of TB active case finding strategies in Cambodia. We will collect baseline information using a questionnaire to inform the evaluation of the interventions.

1. **What is the purpose of this biomedical research?**

This main trial aims to 1) evaluate the effectiveness of an active case finding (ACF) strategy using a seed-and-recruit model for increasing TB case notification in Cambodia; 2) establish the effect of ACF strategies on TB treatment outcomes; 3) evaluate the cost-effectiveness (costs per TB case notified) of different ACF strategies. We will collect baseline data from the questionnaire that we are going to administer to better evaluate the impact of the case finding interventions. This information sheet provides you with information about the study. The principal investigator/researcher will also describe this research to you and answer all your questions. Read the information below and ask questions about anything you don’t understand before deciding whether to take part.

1. **What are the possible risks, discomforts, or inconveniences to me if I participate in this research?**

There are no perceivable risks in this study. Participation is purely voluntary, and the decision not to partake in this study will not have any negative consequences. The survey does not include any specific medical procedures. Any procedures involved in the diagnosis and management of TB during the implementation of TB ACF is in accordance with the standard clinical practice guideline of Cambodia and will be performed by trained staff.

1. **What benefits can I expect from participating in the research?**

There is no direct benefit to you by participating in this study. However, the information provided is important to guide the development of interventions to find undiagnosed TB cases in the community.

1. **Are there any alternative procedures or treatments available to me? What are the potential benefits and risks of such alternatives?**

There are no alternative procedures or treatments available to you, as the survey does not include any medical interventions. The TB case finding strategies are models of care to find undiagnosed cases in the community. The screening, diagnostics tests, and the TB treatment that you will receive are per the standard protocol in Cambodia. The procedures will be the same outside a research setting.

1. **If I am injured as a result of participating in this research, what are the compensation and treatments available to me?**

There are no major perceivable risks in this study. The potential psychological distress because of the survey questions that the study poses are minimal. Participation is purely voluntary, and the decision to not participate in this study or terminate their participation at any point during the study will not have any negative consequences. All screening and diagnostic procedures included as part of the case finding strategies will be conducted by trained and qualified staff at the health centers and/or staff dispatched by the National Centre for Tuberculosis and Leprosy Control. The TB screening, diagnosis, and treatment procedures will be managed according to the standard protocol in Cambodia.

1. **Do I have to incur any expenses by participating in this research?**

No expenses will be incurred by participating in this research.

1. **What will happen to the biological material taken from me?**

Biological materials taken from you are required as per the national guidelines for TB management. Your sputum samples will be subjected to microscopy and GeneXpert tests for TB diagnosis. Finger prick blood tests for HIV will be conducted using point-of-care testing. HIV test is part of the national guideline for TB management, and it will be conducted accordingly. All procedures will be conducted by trained and qualified staff. You will be notified of the results, and referrals for care will be made if necessary. All biological samples will be destroyed after the tests are done. No biological materials will be taken from you for the baseline and follow-up survey.

1. **Will my participation in this research involve the use of any information that will identify me?**

Your personal data (i.e., name, mobile phone number, and address) will be collected in this study for retrieving data from the TB register at the health centers. We will also contact you for the follow-up survey after the 6-months treatment period.

1. **How will my personal identifiers collected from me be kept confidential?**

Research data will be coded, and we will treat your responses with confidentiality. The database will be kept confidential and will not be made publicly available. No one will have access to the database except the research team. Your name and contact information will only be used to contact you for surveys, and it is only available to the research staff. Your address will also be obtained in the reimbursement acknowledgment sheet which would be forwarded to the NUS Finance Department. Your personal data will be discarded after the publication of the results. Consent forms which bear your name will be stored for 10 years. It will not be used in the analysis, and publications/presentation related to this study. Demographic information and data will be archived for a period of 10 years, in accordance with the University’s Research Data Management Policy.

1. **Will any identifiable information obtained from me be used for future biomedical research?**

If you consent, your personal data (i.e., name, mobile phone number, and address) will be retained during this study period so that we can contact you for the exit interview at the end of your treatment period. Your personal data will not be used for other biomedical research activities other than this.

1. **Will I be re-identified in the event of incidental finding(s) arising during the biomedical research?**

No additional medical tests will be performed on you in this study. Other non-biomedical data are survey data which you will provide. Hence, we do not anticipate any incidental findings in this study.

1. **Under what circumstances will I be re-contacted for further consent?**

There are no anticipated circumstances where further consent is required in this study.

1. **Can I withdraw my consent to the research at any time?**

Your participation in this research is voluntary, and it is completely up to you to join the study. You have the right to refuse to answer any specific questions. You can withdraw from the research at any time without giving any reasons, by informing the principal investigator verbally or in writing. Please note that the withdrawal of consent does not affect the research information obtained before the consent is withdrawn, and such information may still be retained and used for research. However, as all research data will be anonymized after the survey, it is not possible to identify research data specific to you. Hence, it is not possible to discard your data should you decide to withdraw after the survey has been conducted. All anonymized data will be analyzed. There will be no penalties or damages imposed on you should you withdraw your consent to participate in this research. Medical care and services that you are receiving from the health center would not be affected, if you choose not to participate in this study.

**Consent Form for Research Subjects**

**Protocol title:**

Effectiveness of an active case finding strategy with a seed-and-recruit model to increase tuberculosis case detection in Cambodia: a cluster randomised controlled trial

**Principal Investigator with the contact number and organization:**

Dr Siyan Yi

*Senior Research Fellow*

Saw Swee Hock School of Public Health

Tahir Foundation Building, National University of Singapore

12 Science Drive 2, Singapore 117546

[ephsyi@nus.edu.sg](mailto:ephsyi@nus.edu.sg)

Phone: (65) 6516 4988

I hereby acknowledge that:

1. My signature is my acknowledgment that I have agreed to take part in the above research.
2. I have received a copy of this information sheet that explains the use of my data in this research. I understand its contents and agree that my contact information and personal health information can be collected from my medical records in the health center, used and shared by the researchers and staff for the research study described in this form.
3. I can withdraw from the research at any point of time during the survey by informing the Principal Investigator, and all my data will be discarded. I understand that my data will be anonymized after completion of the baseline and exit interviews. Therefore, if I decide to withdraw from the research after the survey, it is not possible to discard my data, and the anonymized data will be analyzed.
4. I will not have any financial benefits that result from the commercial development of this research.

**________________________ ___________**

Name and Signature (Participant) Date

**________________________ ___________**

Name and Signature (Consent Taker) Date

**For those who cannot read/sign:**

*If you agree to participate, please say so. You will be given a copy of this form to keep for your own records.*
