# Supplementary material for: Effect of community active case-finding strategies for detection of tuberculosis in Cambodia: study protocol for a pragmatic cluster randomized controlled trial
Source: Trials. 2020 Feb 24;21:220. doi: 10.1186/s13063-020-4138-1 (PMC7041270; doi:10.1186/s13063-020-4138-1)
Supplement: Supplementary file 6 — Additional file 6. PRECIS-2 scores for trial domains. [file 13063_2020_4138_MOESM6_ESM.docx]

**PRECIS-2 scores for trial domains**

|  | Domain | Score | Rationale |
| --- | --- | --- | --- |
| 1 | Eligibility criteria | 5 | The interventions are endorsed by the national TB program in Cambodia and have been widely implemented in the country. The eligibility criteria in the trial — presumptive TB exhibiting symptoms of pulmonary TB, extra-pulmonary TB, and other general TB symptoms (see protocol for details) — are the same as they currently are in the TB case finding interventions outside the research setting. |
| 2 | Recruitment path | 5 | Recruitment will be conducted at the health centers — passive case finding — or during the 3 proposed TB active case finding events — active case finding using the seed-and-recruit model, active case finding targeting household and neighborhood contacts, and active case finding targeting the older population (people aged above 55) using mobile screening units. |
| 3 | Setting | 5 | The interventions are endorsed by the national TB program in Cambodia and have been widely implemented in the country. This study will be conducted in settings where these interventions are normally held. In this trial, eight operational districts were purposively selected to include operational districts with high and low incidence to maintain as much balance as possible. The selection was also based on the number of health centers to increase comparability and generalizability of study findings across the groups. |
| 4 | Organization intervention | 5 | Identical organization that carry out these interventions outside the research setting. |
| 5 | Flex of experimental intervention – delivery | 5 | Identical flexibility should the interventions are carried out outside the research setting. |
| 6 | Flex of experimental intervention – adherence | 5 | Identical flexibility should the interventions are carried out outside the research setting. |
| 7 | Follow-up | 5 | We will follow-up participants to evaluate treatment outcome which is a standard practice for all persons who initiated TB treatment in the country. For people with bacteriologically confirmed TB, sputum samples will be assessed at month-2, month-5, and month-6 of TB treatment. For clinicians diagnosed TB (smear-negative TB), sputum samples of the study participants will be assessed two months after treatment initiation. Other relevant clinical signs and symptoms will be monitored too. Similarly, the follow-up of people with extra-pulmonary TB is based on clinical observations, treatment adherence, and side effects of anti-TB medicines. Health providers at public health facilities will perform the evaluation of sputum samples and other clinical observations in accordance with the national TB guideline. In general, the treatment outcomes of people with pan-TB will be ascertained 6-months after treatment initiation. |
| 8 | Outcome | 4 | The trial primary endpoints are relevant for policy making and program planning. The relevance at an individual-level is relatively minimal. However, individuals would have benefited from early TB diagnosis and treatment. |
| 9 | Analysis | 5 | Intention to treat analysis. |
